# Investigating LST evolution and heatwave patterns using machine learning in the Beijing-Tianjin-Hebei Urban agglomeration

**DOI:** 10.1016/j.isci.2026.116545

**Published:** 2026-06-26

**Authors:** Chen Liu, Maomao Zhang

**Affiliations:** 1 College of Art, Hebei GEO University, Shijiazhuang 050000, China; 2 School of Architecture and Urban Planning, Huazhong University of Science and Technology, Wuhan 430079, China; 3 College of Public Administration, Huazhong University of Science and Technology, Wuhan 430079, China; 4 College of Urban and Environmental Sciences, Central China Normal University, Wuhan 430079, China

**Keywords:** land use, land surface temperature, LST, heat waves, spatial statistics, XGBoost-SHAP model, urban agglomeration

## Abstract

Rapid urbanization and persistent climate warming are jointly intensifying summer thermal risks in large urban agglomerations, with increasingly pronounced coupling between daytime heat exposure and insufficient nighttime cooling. Using MODIS land surface temperature (LST) data, heatwave metrics, Moran’s I and Getis-Ord Gi∗ statistics, and XGBoost-SHAP interpretation, we examined daytime and nighttime thermal evolution across the Beijing-Tianjin-Hebei urban agglomeration (BTHUA) from 2000 to 2024. Daytime LST showed weak net warming with strong interannual fluctuations, whereas nighttime LST increased steadily by 2.3°C. Pixels exposed to compound day-night heatwaves expanded from 6.2% to 18.6%, with persistent hotspots in the southern urban belt and coldspots in northern mountains. SHAP results indicate that elevation exerted the strongest cooling association, while vegetation and albedo mainly shaped daytime LST and nighttime lights and built-up land shaped nighttime LST. These findings may support time-specific and regionally differentiated heat-risk governance.

## Highlights

• Compound day-night heatwave exposure tripled from 2000 to 2024 in the BTHUA
• Persistent thermal hotspots were concentrated in southern urban areas
• Daytime and nighttime LST were shaped by different dominant drivers

## Graphical abstract

**Figure:**
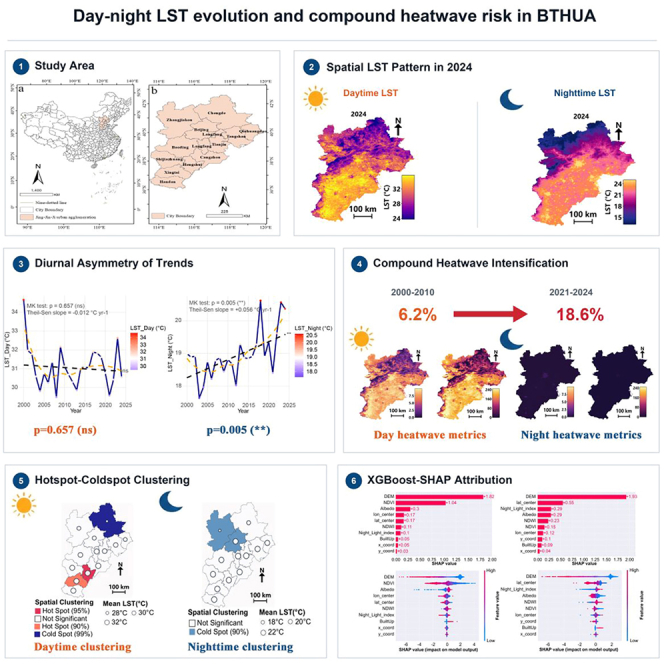

## Teaser

Physics; Applied sciences; Artificial intelligence.

## Introduction

The trend of global warming has become increasingly pronounced, emerging as a critical global environmental issue.^1^^,^^2^^,^^3^ According to the latest report from the World Meteorological Organization (WMO), since the Industrial Revolution, the global average land surface temperature (LST) has risen by approximately 1.1°C, and this upward trend is expected to continue in the future.^4^^,^^5^^,^^6^ Meanwhile, as global urbanization accelerates, the urban heat island effect (UHI) has become increasingly pronounced, emerging as a significant issue in urban environments.^7^^,^^8^ In cities, the proliferation of impermeable hard surfaces (e.g., roads, plazas, and building roofs) and the increased thermal capacity of buildings have led to a narrowing of the daily temperature difference between day and night, resulting in a significant decline in nighttime cooling efficiency.^9^^,^^10^^,^^11^^,^^12^ These changes have significantly increased the risk of heat stress for urban residents at night, particularly during the summer high-temperature season.^13^^,^^14^ Therefore, conducting precise monitoring and in-depth analysis of the spatiotemporal evolution of urban thermal environments and their driving mechanisms is of utmost importance for enhancing urban climate resilience.^15^^,^^16^^,^^17^

Remote sensing technology, as an advanced geographic information technology, has become an indispensable tool in urban LST research due to its significant advantages, including wide coverage, high resolution, and convenient data acquisition.^18^^,^^19^^,^^20^^,^^21^^,^^22^ In early studies, Landsat TM/ETM+ data, with its high spatial resolution, were widely used in urban thermal environment research.^23^^,^^24^^,^^25^^,^^26^ These studies primarily focused on identifying urban heat island patches and assessing the cooling effects of green spaces.^27^^,^^28^^,^^29^^,^^30^^,^^31^ Through these studies, we gained an initial understanding of the spatial distribution characteristics of urban thermal environments, laying a solid foundation for subsequent in-depth research.^32^^,^^33^^,^^34^^,^^35^ However, as research progressed, the limitations of the temporal resolution of Landsat TM/ETM+ data became increasingly apparent. Fortunately, with the emergence of the MODIS MOD11A2 product, its higher temporal resolution has quickly made it the mainstream choice for LST monitoring at regional and national scales.^36^^,^^37^^,^^38^^,^^39^^,^^40^ In recent years, studies based on MODIS or Landsat have improved understanding of urban thermal dynamics at the scale of individual cities or urban agglomerations.^41^^,^^42^^,^^43^^,^^44^ However, the limitations of previous studies remain more specific than generally acknowledged. First, many studies adopt short or discontinuous observation periods, which are sufficient for describing thermal conditions in specific years or seasons but are less capable of detecting staged transitions, persistence, or hotspot consolidation over multi-decadal urbanization.^45^^,^^46^^,^^47^^,^^48^ Second, daytime and nighttime LST are often examined separately, which limits understanding of diurnal asymmetry and the coupled evolution of day-night heat risk.^4^^,^^49^^,^^50^ Third, heat-related assessments are frequently simplified to temperature level or event occurrence, whereas the frequency, duration, severity, and intensity of heatwaves are rarely evaluated within one integrated framework.^6^^,^^51^^,^^52^^,^^53^^,^^54^ Fourth, although spatial statistical tools such as Moran’s I or hotspot analysis are available, they are seldom combined with long-term LST analysis to trace the persistence, expansion, or migration of thermal hotspots and coldspots.^53^^,^^55^^,^^56^^,^^57^ Finally, driver analyses still rely largely on conventional correlation or regression approaches, which are not well suited to capturing nonlinear relationships or the potentially different controls on daytime versus nighttime LST. These unresolved issues make it difficult to build a coherent explanation of how urban thermal patterns evolve over time and why their dominant controls vary across space and between day and night.

Therefore, to gain a more comprehensive understanding of the spatiotemporal evolution of urban thermal environments and their driving mechanisms, it is necessary to move beyond short-term observations and adopt a long-term perspective. The scientific value of an extended study period lies not only in describing whether warming has occurred but also in identifying whether thermal change exhibits phase transitions, path dependence, or persistent hotspot consolidation over time.^55^^,^^56^^,^^57^^,^^58^ A multi-decadal record can better distinguish structural warming signals from short-term climate anomalies and provides a more robust basis for comparing daytime and nighttime trajectories, tracking the emergence of compound heat risks, and revealing whether dominant drivers remain stable or shift during urbanization.^5^^,^^59^^,^^60^

The Beijing-Tianjin-Hebei urban agglomeration (BTHUA) is one of China’s most important urban agglomerations, with a population exceeding 110 million and a GDP accounting for approximately 20% of the national total. It is also a “hotspot” for summer heatwaves and air pollution. Previous studies have documented annual or seasonal LST patterns in the BTHUA and have examined the effects of vegetation, topography, and urban land-use composition on regional thermal conditions.^5^^,^^51^^,^^57^^,^^58^^,^^61^ However, three limitations remain particularly important for this region. First, most BTHUA studies still emphasize daytime thermal patterns, whereas nighttime LST, which is more directly associated with accumulated heat stress and reduced nocturnal recovery, has received much less systematic attention.^59^^,^^62^^,^^63^^,^^64^ Second, existing analyses generally do not connect long-term LST trajectories with multidimensional heatwave characteristics, making it difficult to determine whether regional warming is accompanied by changes in heatwave frequency, duration, severity, and intensity.^18^^,^^65^^,^^66^^,^^67^ Third, the drivers of thermal change in the BTHUA are often interpreted using conventional regression or single-factor comparisons, which limits the identification of nonlinear and potentially divergent controls on daytime and nighttime heat.^44^^,^^68^^,^^69^^,^^70^ These gaps are especially important in a large urban agglomeration such as the BTHUA, where topographic gradients, uneven urban expansion, and strong urban-rural contrasts may produce markedly different thermal mechanisms across space and between day and night.

To address these gaps, this study integrates long-term observation with day-night comparative analysis. Specifically, by examining summer daytime and nighttime LST across 2000–2024, the study evaluates not only the magnitude of warming but also the added scientific value of the long temporal window, namely the ability to detect staged evolution, persistent spatial clustering, and long-term driver differentiation that cannot be captured by short-period assessments. On this basis, spatial autocorrelation methods and the XGBoost-SHAP framework are combined to link thermal evolution patterns with their dominant natural and urban drivers. Through the organic integration of the aforementioned research methods, this study aims to provide a comprehensive and systematic research framework for urban thermal environment studies in the BTHUA and other urban regions, thereby better understanding and addressing the challenges posed by changes in the urban thermal environment.

## Results

### Spatiotemporal distribution of LST_Day

**Figure 1:**
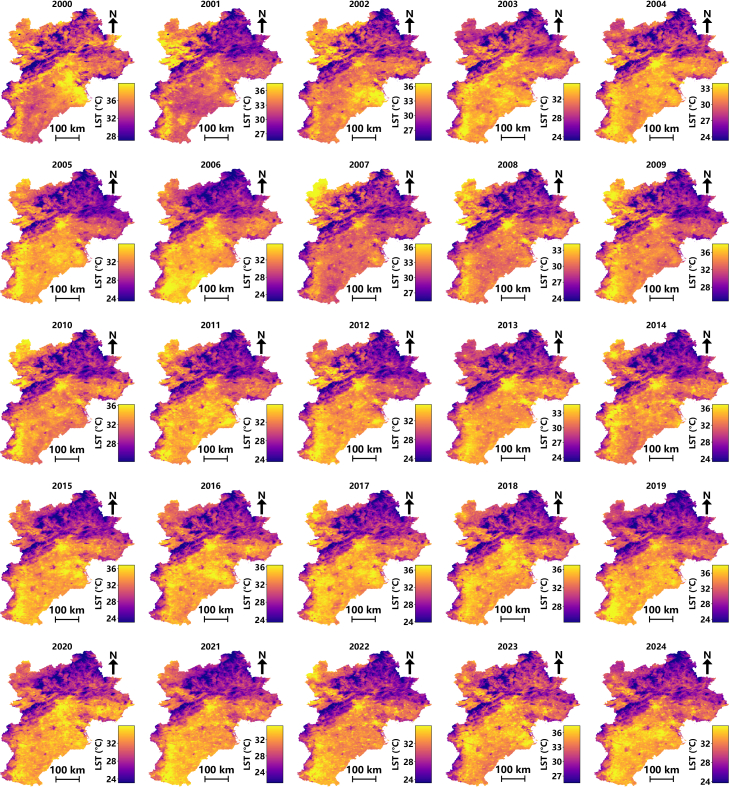
Spatio-temporal distribution of LST_Day

**Figure 2:**
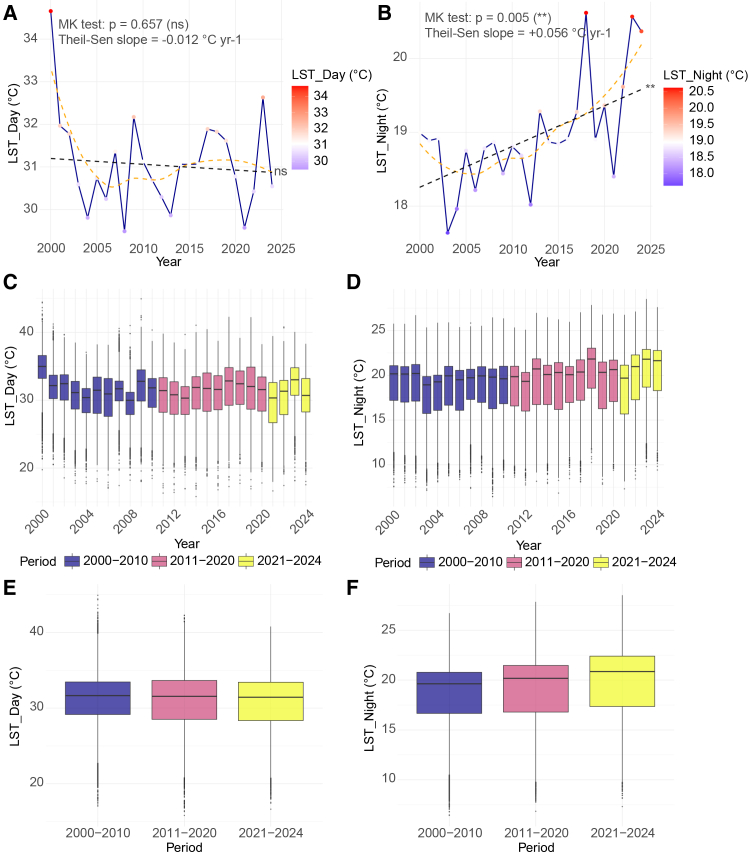
Interannual trends and phase statistics of LST_Day and LST_Night (A) Mann-Kendall test and Theil-Sen slope for daytime LST. (B) Mann-Kendall test and Theil-Sen slope for nighttime LST. (C) Annual distribution of daytime LST. (D) Annual distribution of nighttime LST. (E) Phase-based distribution of daytime LST. (F) Phase-based distribution of nighttime LST.

Figure 1 presents the inter annual spatial distribution of summer LST_Day in the BTHUA from 2000 to 2024. The maps more clearly show a stable south-north and plain-mountain thermal gradient, with relatively high daytime LST concentrated in the central and southern plains and relatively low LST persisting in the Taihang-Yanshan mountain system and the northern mountainous counties. In spatial terms, the Beijing-Tianjin metropolitan region and the urbanized belt extending into southern Hebei remain the main daytime warm zones, whereas Zhangjiakou, Chengde, and other higher-elevation areas consistently act as regional cool areas. In temporal terms, daytime LST does not increase monotonically, but instead exhibits clear interannual fluctuations superimposed on an overall warming trajectory. Compared with the early study period, warm patches became broader and more connected after 2010, and the areal extent of high-LST pixels remained large in the late study period, especially in warm years such as 2019 and 2022. This spatial interpretation is supported by the quantitative results in Figures 2A, 2C, and 2E, which indicate that daytime LST was characterized by strong interannual and phase-related variability. Although the fitted trend in Figure 2A suggests a weak net increase over the 25-year period, the distributional and phase statistics in Figures 2C and 2E show that daytime LST did not increase monotonically and even declined during some phases. Therefore, daytime thermal change in the BTHUA should be interpreted as fluctuation dominated rather than as a steady warming process. Overall, summer LST_Day increased by about 1.4°C during 2000–2024, indicating that daytime warming in the BTHUA is characterized by phased fluctuation rather than simple linear growth.

### Spatiotemporal distribution of LST_Night

**Figure 3:**
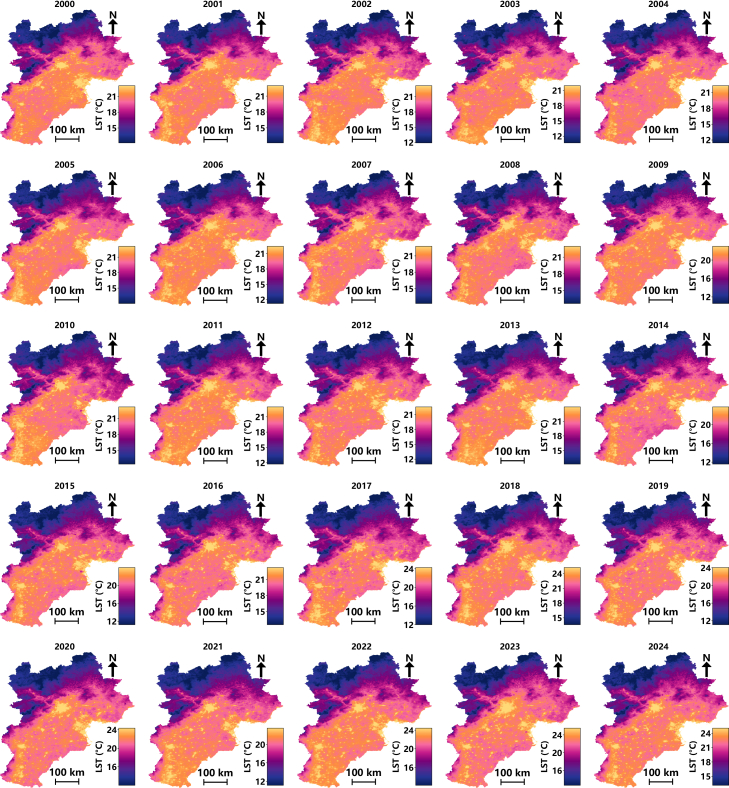
Spatial distribution of LST_Night

**Figure 4:**
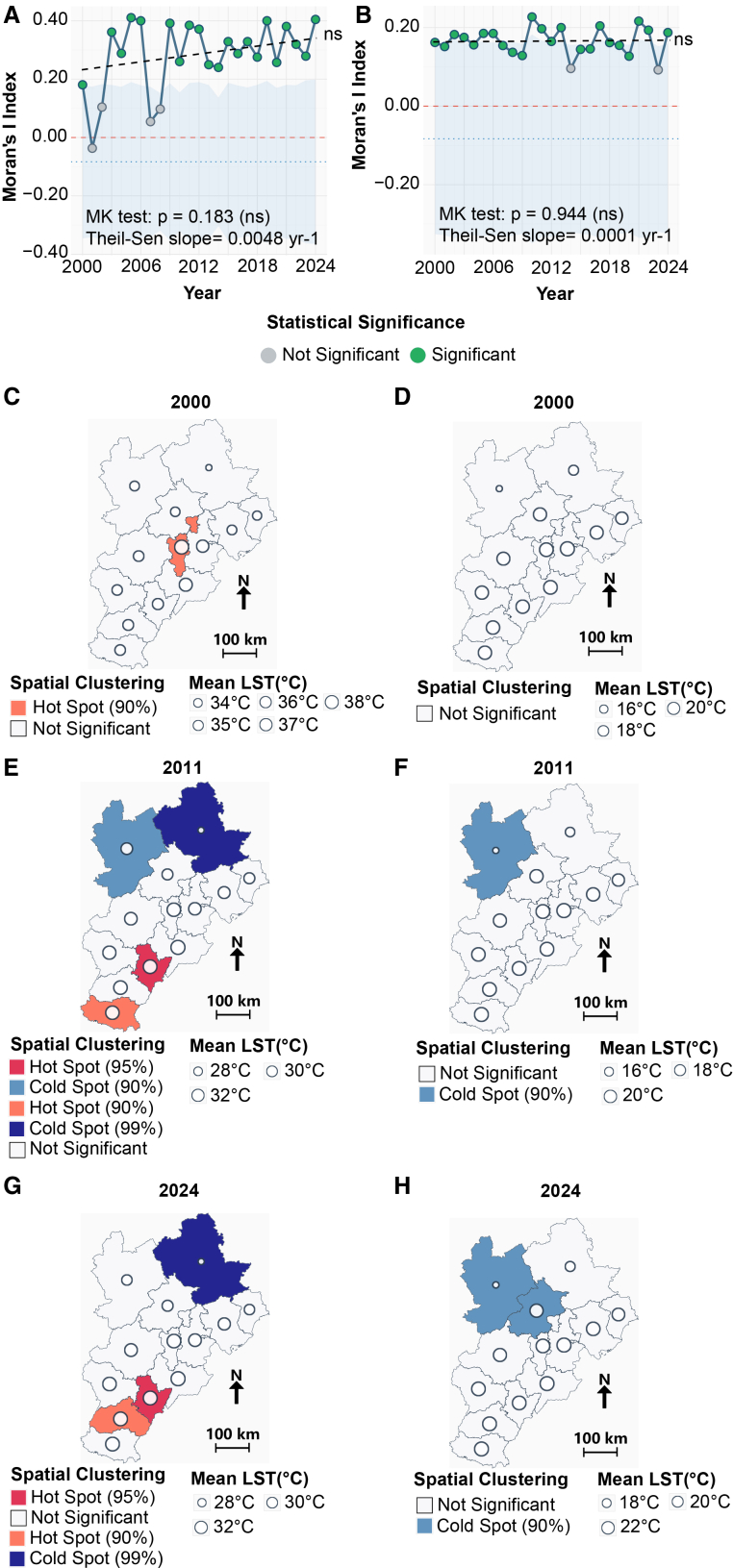
Global spatial autocorrelation and local clustering patterns of LST (A) Interannual variation in Moran’s I for daytime summer LST. (B) Interannual variation in Moran’s I for nighttime summer LST. (C) Daytime LST hotspot and coldspot pattern in 2000. (D) Nighttime LST hotspot and coldspot pattern in 2000. (E) Daytime LST hotspot and coldspot pattern in 2011. (F) Nighttime LST hotspot and coldspot pattern in 2011. (G) Daytime LST hotspot and coldspot pattern in 2024. (H) Nighttime LST hotspot and coldspot pattern in 2024.

Figure 3 shows the spatiotemporal distribution of summer LST_Night in the BTHUA from 2000 to 2024. Compared with the daytime pattern, the nighttime maps display a clearer urban-rural contrast and stronger persistence of warm patches. Low nighttime LST remains concentrated in the Taihang and Yanshan mountains and in northern areas such as Zhangjiakou and Chengde, highlighting the cooling role of elevation and topography. By contrast, higher LST_Night is stably distributed in the Beijing-Tianjin metropolitan area and southern Hebei, where built-up surfaces and nighttime human activities favor heat storage and delayed cooling after sunset. In temporal terms, nighttime warming is more coherent than daytime warming: warm areas gradually expand from scattered urban cores to a broader southern urban belt, and high-temperature patches become more continuous in the later years. This interpretation is further supported by Figures 4B, 4D, and 4F, which show a steadier interannual increase in LST_Night and a more pronounced upward concentration of the nighttime thermal distribution in the later period. Over 2000–2024, LST_Night rose by about 2.3°C, exceeding the daytime increase and indicating an intensifying nocturnal urban heat island effect.

### Interannual trends and phased evolutionary characteristics of LST

To complement the spatial patterns shown in Figures 1 and 3, Figure 2 summarizes the interannual trends and distributional characteristics of summer LST in the BTHUA from 2000 to 2024 for daytime (Figures 2A, 2C, and 2E) and nighttime (Figures 2B, 2D, and 2F). The results reveal a clear diurnal asymmetry in the temporal evolution of LST. For daytime LST, although the fitted curve in Figure 2A shows short-term fluctuations and a slight recovery after the mid-2000s, the Mann-Kendall test (MK) indicates that the overall monotonic trend is not statistically significant (*p* ≥ 0.05, ns). The corresponding Theil-Sen slope is also small, suggesting that daytime LST should not be interpreted as exhibiting a robust long-term warming trend over the full study period. This interpretation is further supported by Figures 2C and 2E, which show pronounced interannual and phase-related variability rather than a consistent upward shift. By contrast, nighttime LST shows a statistically significant increasing trend in Figure 2B, as confirmed by the MK test (*p* = 0.005), together with a positive Theil–Sen slope. Figures 2D and 2F further indicate an upward shift in the central tendency and upper-tail distribution of nighttime LST across the three periods. These results suggest that daytime thermal change in the BTHUA was mainly fluctuation-dominated, whereas nighttime warming was more persistent and statistically robust. Overall, the intensified nighttime warming implies a weakening of nocturnal cooling capacity and increased cumulative heat retention, which may contribute to the amplification of regional heat risk.

### Spatial autocorrelation of LST and hotspot evolution characteristics

Figure 4 shows the global spatial autocorrelation of summer LST_Day and LST_Night (Figures 4A and 4B) and the local spatial clustering patterns in typical years (2000, 2011, and 2024) (Figures 4C–4H) from 2000 to 2024, aiming to reveal the spatial clustering characteristics of the regional thermal environment and their temporal stability. At the global scale, LST_Day generally exhibits positive spatial autocorrelation during most years, indicating that daytime thermal conditions were not randomly distributed but showed a certain degree of spatial clustering. However, the MK test suggests that the interannual change in Moran’s I is not statistically significant (*p* = 0.183, ns), although the Theil-Sen slope is slightly positive. Therefore, the daytime spatial clustering pattern should be interpreted as relatively persistent but not as a statistically robust increasing trend. For LST_Night, Moran’s I remains positive but weaker overall, and the MK test also indicates no significant monotonic trend over the study period (*p* = 0.944, ns), with an almost zero Theil-Sen slope. This result suggests that nighttime spatial autocorrelation was relatively stable and did not show clear evidence of progressive spatial concentration. At the local scale, the hotspot maps for 2000, 2011, and 2024 reveal a more spatially explicit pattern of thermal differentiation. The southern urbanized belt repeatedly appears as a hotspot area, whereas the northern mountainous region tends to act as a persistent coldspot zone, particularly in 2011 and 2024. Compared with 2000, the spatial contrast between southern hotspots and northern coldspots becomes visually clearer in the later typical years; although, the global Moran’s I trend analysis indicates that this change should be interpreted cautiously as a pattern of spatial persistence rather than a statistically confirmed intensification. Overall, Figure 4 demonstrates that the BTHUA has maintained a differentiated summer thermal structure characterized by recurrent southern hotspots and northern cold sources. This spatial configuration provides important support for refined heat-risk zoning, targeted mitigation in urbanized hotspot areas, and the conservation of mountainous cold-source regions.

### Spatiotemporal characteristics of heatwave events

**Figure 5:**
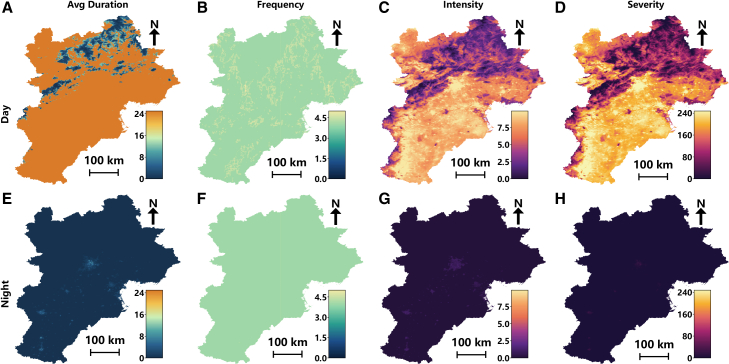
Spatial distribution of daytime and nighttime summer heatwave events (A) Daytime heatwave average duration. (B) Daytime heatwave frequency. (C) Daytime heatwave intensity. (D) Daytime heatwave severity. (E) Nighttime heatwave average duration. (F) Nighttime heatwave frequency. (G) Nighttime heatwave intensity. (H) Nighttime heatwave severity.

Building on the long-term evolution of daytime and nighttime LST and the increasing concentration of high-LST clusters, Figure 5 presents the spatial patterns of four heatwave metrics—frequency, average duration, severity, and intensity—for daytime (Figures 5A–5D) and nighttime (Figures 5E–5H). The figure shows that daytime heatwaves are spatially broader, whereas nighttime heatwaves are more spatially concentrated in the major urban cores. For daytime conditions, relatively high duration, severity, and intensity are mainly distributed in the Beijing-Tianjin-Hebei plain, especially in the southern urbanized belt, indicating strong persistence of accumulated heat stress in low-elevation built-up areas. Nighttime heatwave metrics are generally weaker in absolute magnitude, but they cluster more clearly in Beijing, Tianjin, and adjacent metropolitan zones, suggesting that urban heat storage and delayed nocturnal cooling are key mechanisms of nighttime risk. When daytime and nighttime patterns are read together, the figure also supports the emergence of compound day-night thermal stress, with persistent urban cores experiencing repeated overlap of daytime and nighttime heatwave conditions. This interpretation is consistent with the marked increase in the proportion of day-night compound heatwave pixels, from 6.2% in 2000 to 18.6% in the later period. Overall, the heatwave maps indicate that regional heat risk in the BTHUA is evolving from relatively isolated daytime extremes toward more persistent and spatially concentrated day-night thermal stress. To further interpret the spatial background of these thermal patterns, the following section provides a representative-year SHAP-based attribution analysis for daytime and nighttime LST, rather than a temporally matched explanation of long-term heatwave evolution.

### Representative-year spatial attribution of daytime and nighttime LST based on XGBoost-SHAP

**Figure 6:**
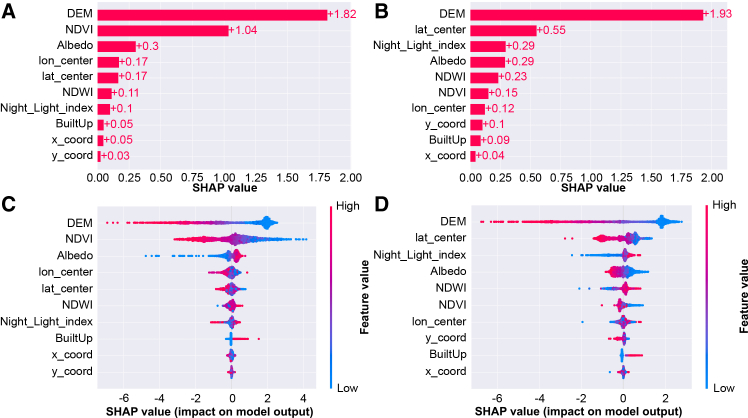
SHAP results of the main drivers of LST (A) Mean absolute SHAP values for the daytime LST model. (B) Mean absolute SHAP values for the nighttime LST model. (C) SHAP summary plot for daytime LST. (D) SHAP summary plot for nighttime LST.

It is important to note that the XGBoost-SHAP analysis is conducted using data from the year 2020. This analysis is designed to explain the spatial heterogeneity of LST patterns within a representative summer, rather than to model the temporal evolution of heatwaves over the 25-year period (2000–2024). While the long-term trends indicate a warming trajectory, this section focuses on identifying the landscape and urban factors that govern the spatial distribution of temperature at a specific point in time. Figure 6 presents the XGBoost-SHAP models for the representative year 2020 and summarizes the relative contributions and association patterns of multiple factors with predicted LST_Day and LST_Night in the BTHUA. This analysis is intended to explain representative-year spatial heterogeneity in LST rather than the full interannual evolution of heatwave metrics. Figures 6A and 6B show the mean absolute SHAP values for LST_Day and LST_Night, respectively, while Figures 6C and 6D present the corresponding SHAP distribution plots, illustrating how the contributions of each variable to the model output vary across different feature values. In the LST_Day model (Figure 6A), Digital Elevation Model (DEM) shows the largest mean absolute SHAP value (+1.82), suggesting that elevation is the variable most strongly associated with lower predicted daytime LST in the model. Normalized Difference Vegetation Index (NDVI) (+1.04) and Albedo (+0.30) also show relatively strong associations with lower predicted LST_Day. Other factors such as lon_center, lat_center, Normalized Difference Water Index (NDWI), water body index, Night_Light_index, and BuiltUp make comparatively smaller contributions to the model output. In the LST_Night model (Figure 6B), DEM also has the largest mean absolute SHAP value (+1.93), indicating a strong model-based association with lower predicted nighttime LST. Latitude (lat_center) and Night_Light_index (both +0.29) make the next largest contributions, suggesting that the spatial pattern of nighttime LST is more closely associated with locational and urbanization-related variables than in the daytime model. The SHAP distribution plots (Figures 6C and 6D) further show the direction and nonlinearity of these model-based associations. For example, in the daytime model, high DEM and NDVI values are generally associated with negative SHAP values, indicating lower predicted LST_Day, whereas high BuiltUp and Night_Light_index values are more often associated with positive SHAP values, indicating higher predicted LST. In the nighttime model (Figure 6D), high Night_Light_index values and some high Albedo values are also associated with positive SHAP responses. Overall, the SHAP results for the representative year suggest that topographic variables are most strongly associated with lower predicted LST in both daytime and nighttime models, while ecological variables such as vegetation and Albedo are more strongly associated with the daytime model, and urbanization-related variables are more prominent in the nighttime model. These findings should be interpreted as representative-year spatial associations rather than temporally comprehensive explanations of the 2000–2024 LST and heatwave evolution.

## Discussion

### Diurnal asymmetry of regional warming

The observed diurnal asymmetry does not imply that daytime LST increased steadily throughout the study period. Instead, daytime LST was more strongly influenced by interannual climatic variability, vegetation conditions, surface moisture, and short-term atmospheric background, resulting in a fluctuation-dominated temporal pattern. In contrast, nighttime LST showed a more persistent warming tendency, which is more closely related to heat storage in built-up surfaces, delayed nocturnal heat release, and reduced nighttime cooling efficiency. Therefore, the key thermal signal in the BTHUA is not simply an overall increase in both daytime and nighttime LST, but a stronger and more stable nocturnal warming process.

This day-night divergence also implies that the warming process is not only a matter of higher temperature values but also of changing thermal behavior across the daily cycle. Daytime warming is more sensitive to interannual climatic variability and short-term atmospheric conditions, whereas nighttime warming more directly reflects the cumulative effects of urban form, surface properties, and anthropogenic disturbance.^71^^,^^72^ From a risk perspective, this distinction is critical because the loss of nighttime cooling reduces opportunities for physiological recovery, increases cooling-energy demand, and may intensify the long-term burden of heat exposure on vulnerable populations.^73^^,^^74^ Therefore, the stronger persistence of nighttime warming should be understood as a structural signal of changing regional thermal risk rather than merely a secondary feature of daytime heat accumulation.

### Evolution of compound heatwave risk

The main value of the heatwave analysis lies in showing that long-term warming in the BTHUA is being translated into a more complex and persistent risk structure, rather than simply producing higher background temperatures. In particular, the increase in day-night compound heatwave exposure indicates that urban heat stress is no longer dominated by isolated daytime extremes but is increasingly characterized by the coupling of daytime exposure and nighttime non-recovery. This transition is especially important because it changes the nature of thermal risk from episodic stress to cumulative stress across the full daily cycle.

Such a shift has broader implications for understanding urban climate vulnerability. When high daytime temperatures are followed by elevated nighttime LST, the human body, buildings, and urban infrastructure are exposed to prolonged rather than intermittent thermal loading. In large urban agglomerations, this process may amplify health risks, increase pressure on energy systems, and deepen inequalities in access to cooling resources. Therefore, the significance of compound heatwaves lies not only in their greater extent but also in the fact that they reveal a transition in the dominant form of urban heat risk. This finding suggests that heat governance should move beyond daytime-centered intervention and adopt a full-cycle framework that considers daytime exposure, nighttime recovery, and the spatial differentiation of vulnerability.^73^^,^^74^^,^^75^

### Consolidation of hotspot-coldspot structure

The spatial autocorrelation results suggest that the thermal landscape of the BTHUA is becoming increasingly organized and path dependent. As hotspot clustering intensifies, high-temperature areas appear to be more firmly anchored within the southern urbanized belt, while coldspots remain persistently associated with the northern mountainous region. This pattern implies that the regional thermal structure is not fluctuating randomly from year to year but is gradually stabilizing under the combined influence of topography, urban expansion, and land-surface transformation.

The persistence of this hotspot-coldspot differentiation is important from both a physical and planning perspective. Once high-temperature areas are reinforced by concentrated development, dense impervious surfaces, and continuous urban functional agglomeration, they are less likely to revert to cooler states without substantial structural intervention. By contrast, the long-term stability of coldspots indicates that mountainous terrain, ecological land cover, and regional ventilation conditions continue to provide a buffering effect against heat accumulation.^76^^,^^77^ This spatial polarity suggests that future heat-risk management should not focus solely on mitigating existing hotspots but should also protect mountain cold sources, ecological cooling spaces, and other areas that contribute to the regional thermal balance. In this sense, spatial thermal governance should be understood as both a mitigation strategy and a conservation strategy.^78^^,^^79^

### Diurnal differentiation of thermal drivers

The SHAP results indicate that daytime and nighttime thermal patterns are governed by partially different process chains. During the day, ecological and surface-radiation-related factors play a stronger role because they directly influence energy partitioning, evapotranspiration, and surface heating efficiency. At night, however, urbanization-related factors become more prominent, suggesting that heat storage, anthropogenic activity, and delayed release from built surfaces are more important in shaping nocturnal thermal conditions.^74^^,^^80^ This day-night contrast helps explain why thermal mitigation cannot rely on a single intervention logic across the full daily cycle.

From a planning perspective, the distinction between daytime and nighttime drivers implies that different regulatory priorities are needed for different periods of heat exposure. Daytime mitigation is more closely linked to ecological enhancement, surface reflectivity management, and improvement of land-cover conditions, whereas nighttime mitigation requires greater attention to urban density, built-up intensity, and the thermal retention characteristics of developed land.^81^^,^^82^ These patterns are broadly consistent with previous studies showing that vegetation-related and surface-radiation factors tend to play a stronger cooling role during the daytime, whereas urbanization-related indicators are more closely associated with nighttime heat retention.^49^^,^^79^^,^^83^^,^^84^ However, in the present study, these relationships should be interpreted as model-based spatial associations derived from the SHAP framework rather than direct evidence of causality. Even so, the observed diurnal differentiation provides useful guidance for building a more adaptive urban cooling strategy, one that combines daytime ecological regulation with nighttime control of thermal accumulation and anthropogenic heat-related pressure.

### Limitations of the study

This study contributes to a more integrated understanding of urban thermal dynamics in the BTHUA by linking long-term day-night LST evolution, heatwave characteristics, spatial clustering, and driver attribution within a unified analytical framework. Nevertheless, several limitations remain and provide directions for further research. First, this study mainly relied on MODIS LST products with a spatial resolution of 1 km. Although these data are suitable for examining regional-scale thermal patterns across a large urban agglomeration, they are less capable of capturing fine-scale thermal heterogeneity within urban blocks, street canyons, and specific land-use patches. Future studies should integrate higher-resolution remote sensing data, such as Landsat, Sentinel, and unmanned aerial vehicle observations, to improve the characterization of neighborhood-scale thermal environments. Combining multi-resolution data would also help bridge regional thermal patterns with local planning interventions. Second, the machine-learning attribution analysis was based on explanatory variables compiled for the representative year 2020, whereas the LST and heatwave analyses covered the period from 2000 to 2024. Therefore, the XGBoost-SHAP results should be interpreted as representative-year spatial associations rather than temporally matched explanations of the full 25-year evolution. Future research should construct annual or multi-period datasets of natural, land-use, and socioeconomic drivers to examine whether dominant explanatory patterns remain stable across different stages of warming, urban expansion, and land-surface transformation. Third, this study focused primarily on physical thermal exposure, while social vulnerability and future risk pathways were not fully incorporated.

### Conclusions

This study utilized MODIS LST data from the BTHUA during the summer months from 2000 to 2024, combined with heatwave identification, spatial statistics, autocorrelation analysis, and the SHAP explanation model, to systematically investigate the spatiotemporal evolution characteristics and driving mechanisms of the regional thermal environment. The main conclusions are as follows: the temporal evolution of LST shows clear diurnal asymmetry. Daytime LST exhibited pronounced interannual variability and only a weak net increase suggested by the fitted trend, rather than a clear monotonic warming process. In contrast, nighttime LST increased more steadily, with a cumulative rise of approximately 2.3°C over the 25-year period, reflecting the increasingly prominent nocturnal urban heat island effect and reduced nighttime cooling capacity. However, LST_Night showed a steady increase, with a cumulative increase of 2.3°C and an annual growth of 0.092°C, reflecting the increasingly prominent urban heat island effect. Heatwave events exhibit a trend toward diurnal characteristic differentiation and “complexification”. Daytime heatwaves are characterized by high frequency, intensity, and widespread coverage, while nighttime heatwaves are concentrated in urban heat island areas. The proportion of regions experiencing heatwaves simultaneously during both daytime and nighttime has increased from 6.2% to 18.6%, indicating an expansion of heat disaster risks across all time periods. The spatial pattern of the thermal environment has become increasingly solidified, with the “hot south, cold north” pattern remaining stable over the long term. High-temperature zones are concentrated in the Beijing-Tianjin and central-southern Hebei urban clusters, while coldspots are locked in northern high-altitude regions such as Zhangjiakou and Chengde. The global Moran’s I value increased by 41.4% between 2000 and 2024, reflecting enhanced spatial clustering. In the representative year 2020, the SHAP-based attribution results indicate clear diurnal differences in spatial associations: daytime LST is more closely associated with ecological factors such as NDVI and Albedo, whereas nighttime LST is more strongly associated with urbanization indicators such as BuiltUp and Night_Light_index. DEM remains the dominant cooling factor both day and night, indicating that thermal environment management must be tailored to the time of day. Urban thermal risk management should adopt a coordinated regulatory approach that integrates time-segmented, region-specific, and multi-factor strategies. It is recommended to establish a dynamic regulatory mechanism centered on “nighttime heat source mitigation and daytime green space expansion,” and to focus on “high-temperature hotspot areas” for differentiated management to enhance regional climate resilience.

## Resource availability

### Lead contact

Requests for further information and resources should be directed to and will be fulfilled by the lead contact, Maomao Zhang (zhangmaomao516@126.com).

### Materials availability

This study did not generate new unique materials.

### Data and code availability

• All datasets used in this study are publicly available and can be accessed via the links provided in the key resources table.
• All custom code is available on request from the lead contact.
• Any additional information required to reanalyze the data reported in this paper is available from the lead contact upon request.

## Acknowledgments

We would like to express our sincere gratitude to the editors and reviewers who have put considerable time and effort into their comments on this paper.

## Author contributions

Investigation, data curation, formal analysis, validation, writing – original draft preparation, writing – original draft, C.L.; conceptualization, methodology, investigation, formal analysis, writing – original draft preparation, writing—review and editing, supervision, project administration, M.Z. All authors have read and agreed to the published version of the manuscript.

## Declaration of interests

The authors declare no competing interests.

## STAR★Methods

### Key resources table

**Table:** 

REAGENT or RESOURCE	SOURCE	IDENTIFIER
**Deposited data**		

LST	MOD11A2 (NASA LP DAAC; native 1 km)	https://doi.org/10.5067/MODIS/MOD11A2.061
DEM	NASA SRTMGL1 v003 (NASA LP DAAC; resampled to 1 km)	https://doi.org/10.5067/MEASURES/SRTM/SRTMGL1.003
NDVI	MOD13A2 (NASA LP DAAC; native 1 km)	https://doi.org/10.5067/MODIS/MOD13A2.061
NDWI	MOD09A1 (NASA LP DAAC; resampled to 1 km)	https://doi.org/10.5067/MODIS/MOD09A1.061
Albedo	MCD43A3 (NASA LP DAAC; resampled to 1 km)	https://doi.org/10.5067/MODIS/MCD43A3.061
Nighttime light data	VIIRS Nighttime Lights Annual V2/V2.2 (EOG; resampled to 1 km)	https://eogdata.mines.edu/products/vnl/
Built-up	MCD12Q1 (NASA LP DAAC; resampled to 1 km)	https://doi.org/10.5067/MODIS/MCD12Q1.061

**Software and algorithms**		

ArcGIS 10.8	ESRI	https://desktop.arcgis.com/zh-cn/desktop/index.html
python 3.9.13	python	https://www.python.org/

### Method details

#### Study area

**Figure 7:**
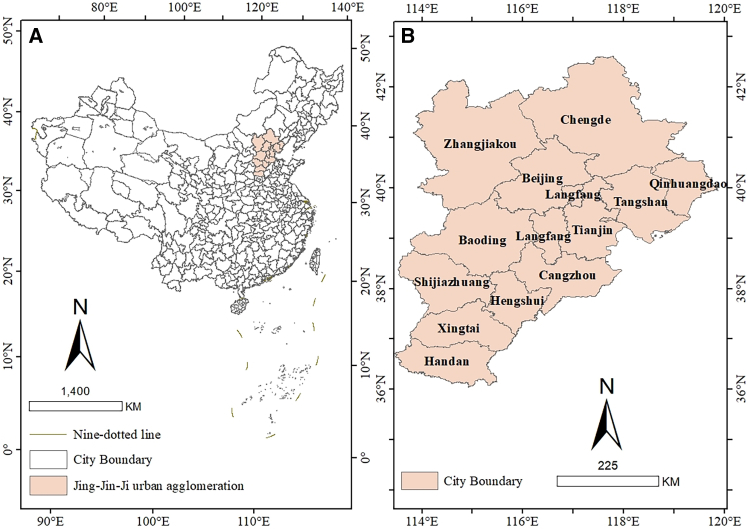
Location distribution map of the BTHUA (A) Location of the BTHUA within China. (B) Administrative units and major cities within the BTHUA.

This study selects the BTHUA as the study area. This region is one of China’s three national-level urban agglomerations, located in North China, with a geographical range roughly between 113°45′ and 119°30′ east longitude and 36°00′ and 42°30′ north latitude (Figure 7). The total area of this region is approximately 218,000 km^2^, with a permanent population exceeding 110 million, making it one of China’s key political, economic, and cultural hubs. The annual average temperature distribution shows a significant north-south gradient, ranging from approximately 5°C–14°C. Additionally, this region is one of the most severely affected by air pollution in China, with urban thermal environment issues being particularly prominent, especially during summer when a distinct UHI effect forms. With the acceleration of urbanization, the impervious surface area (ISA) in this region has expanded rapidly, exerting a significant influence on the regional LST. This study focuses on the summer LST changes in the region from 2000 to 2024, examining their spatiotemporal distribution characteristics and trends, and further exploring the potential mechanisms underlying LST changes driven by urbanization. The study area exhibits significant differences in urban functional levels, encompassing megacities (such as Beijing and Tianjin), numerous medium-sized and small cities, and vast rural areas. This complex urban system structure provides a rich spatial heterogeneity backdrop for research on urban thermal environment changes.

#### Data

The data used in this study (Table 1) served two different analytical purposes. Summer LST data were compiled annually from 2000 to 2024 for the spatiotemporal trend analysis, whereas the explanatory variables used in the XGBoost-SHAP analysis, including DEM, NDVI, NDWI, Albedo, Nighttime Light, and Built-up, were prepared for the year 2020 at a uniform spatial resolution of 1 km to ensure comparability and spatial consistency among variables. Accordingly, the machine-learning analysis was designed to explain the spatial variability of LST in a representative year rather than the full temporal evolution over the 25-year period. Therefore, the SHAP analysis should be understood as a supplementary spatial attribution module for representative-year LST patterns, rather than a direct explanatory model for the long-term evolution of heatwave metrics from 2000 to 2024. Specifically, LST and Albedo data are derived from the MODIS products MCD43A3 and MOD11A2, respectively. NDVI and NDWI are extracted from MOD13A2 and MOD09A1, respectively. Night light data are based on the VIIRS nighttime light remote sensing product. Built-up data are sourced from the MODIS MCD12Q1 product. DEM data are obtained from the SRTM elevation dataset provided by NASA. All data have undergone spatial resampling and regional cropping to align with the geographical scope of the BTHUA.

**Table 1:** Definition and calculation of research data

Variable	Data source	Spatial resolution	Temporal resolution	Time span used in this study
LST	MOD11A2	1 km	8-day	2000–2024 (summer)
DEM	NASA SRTM	1 km	static	baseline terrain data
NDVI	MOD13A2	1 km	16-day	2020 (summer composite)
NDWI	MOD09A1	1 km	8-day	2020 (summer composite)
Albedo	MCD43A3	1 km	daily	2020 (summer composite)
Nighttime light	VIIRS	1 km	annual	2020
Built-up	MCD12Q1	1 km	annual	2020

#### LST data processing

This study uses MODIS remote sensing products (MOD11A2) to obtain LST data with a spatial resolution of 1 km and a temporal resolution of 8 days. To reduce the influence of cloud contamination and low-quality retrievals, the MOD11A2 quality control information was used to exclude unreliable LST pixels before seasonal aggregation. Only valid observations retained after quality screening were used to calculate the annual summer mean LST for each pixel. Because MOD11A2 is an 8-day composite product, the compositing procedure itself also helps reduce short-term cloud effects relative to single-date observations. Pixels with no valid observations during the target period were treated as missing values and were not forcibly interpolated, so as to avoid introducing artificial thermal signals into the long-term analysis. The data covers the period from 2000 to 2024, encompassing 25 full years. We define the summer season as June 1 to August 31 (DOY = 153–244) each year and extract all imagery during this period for processing.

On the Google Earth Engine (GEE) platform, we used the study area vector boundaries for masking and cropping, dividing the data into two bands: “LST_Day” and “LST_Night,” representing daytime and nighttime land surface temperatures, respectively. For each year, we averaged all summer images for that year at the pixel level in a time series to obtain the annual average summer LST^18^^,^^85^:

(Equation 1) $${LST}_{y}^{d/n}=\frac{1}{N_{y}}\sum_{i=1}^{N_{y}}{LST}_{i}^{d/n}$$

Among them, ${LST}_{y}^{d/n}$ represents the average daytime/nighttime surface temperature in year *y*, *N*_*y*_ represents the number of images in summer of that year, and ${LST}_{i}^{d/n}$ represents the LST value in time period *i*. All LST grid data were further resampled, aligned, and stacked to provide a data foundation for subsequent analysis.

#### Heatwave identification and construction of composite heatwave metrics

To capture the spatiotemporal characteristics of extreme high-temperature events, based on World Meteorological Organization (WMO) standards and regional research practices, events with LST values higher than the 90th percentile of the summer of the same year for three consecutive days or more are defined as heatwaves.^86^^,^^87^ First, a temperature distribution set is constructed based on the LST values of all pixels for each year, and the 90% value is extracted as the heatwave threshold:

(Equation 2) $$T_{90}^{y}=Percentile90\left({\left\{{LST}_{j}^{y}\right\}}_{j=1}^{M}\right)$$

Then, construct the following recognition function for each pixel

(Equation 3) $${HW}_{t}=\left\{ \begin{array}{c} 1,if\;{LST}_{t}\geq T_{90}^{y}\wedge {LST}_{t+1}\geq T_{90}^{y}\wedge {LST}_{t+2}\geq T_{90}^{y}\\ 0,otherwise \end{array}\right.$$

For each pixel, let *E*_*y*_denote the set of heatwave events identified in year *y*, and let *n*_*y*_be the total number of events. For the *k-th* event, let *d*_*k*_ denote its duration (number of consecutive observation periods exceeding the threshold), and let *LST*_*k*,*t*_ denote the LST value in period *t* within that event. Let *T*_*y*_ denote the heatwave threshold for year *y*. The four heatwave metrics were defined as follows:

Frequency: the number of heatwave events in year *y*,

(Equation 4) $$E_{y}=n_{y}$$

Average duration: the mean number of consecutive heatwave periods per event,

(Equation 5) $$D_{y}=\frac{1}{n_{y}}\sum_{k=1}^{n_{y}}d_{k}$$

Intensity: the mean exceedance above the threshold during all heatwave periods,

(Equation 6) $$I_{y}=\frac{\sum_{k=1}^{n_{y}}\sum_{t=1}^{d_{k}}\left({LST}_{k,t}-T_{y}\right)}{\sum_{k=1}^{n_{y}}d_{k}}$$

Severity: the cumulative exceedance above the threshold across all heatwave periods,

(Equation 7) $$S_{y}=\sum_{k=1}^{n_{y}}\sum_{t=1}^{d_{k}}\left({LST}_{k,t}-T_{y}\right)$$

This study identifies “daytime heat waves,” “nighttime heat waves,” and “day-night composite heat waves,” A day-night composite heatwave pixel was defined as a pixel that simultaneously satisfied the daytime and nighttime heatwave criteria within the same year.^88^^,^^89^^,^^90^ The proportion of such pixels was used to characterize the spatial extent of compound thermal stress. This process enables multidimensional quantification of regional heat wave evolution, revealing the intensity of extreme heat exposure in cities at different time scales.^71^ Based on the binary heatwave identification for each pixel and each summer observation period, four heatwave metrics were calculated: frequency, average duration, intensity, and severity. In this study, the term “Composite Heatwave Index” does not refer to a single weighted indicator, but to an integrated metric framework that jointly characterizes the occurrence, persistence, and magnitude of heatwave events from multiple dimensions.^91^^,^^92^^,^^93^^,^^94^ Specifically, frequency describes how many heatwave events occurred in a given summer; average duration describes the mean length of these events; intensity describes the average exceedance of LST above the annual heatwave threshold during heatwave periods; and severity describes the accumulated exceedance of LST above the threshold across the full duration of all heatwave events. These four indicators were calculated separately for daytime and nighttime heatwaves and were then mapped to reveal their spatial differences.

#### Spatial aggregation analysis and identification of High-LST areas

To examine the spatial aggregation and significant distribution of high-temperature phenomena, this study introduced Moran’s I spatial autocorrelation index to perform a spatial global aggregation analysis of raster LST data.^78^^,^^95^ The calculation formula is as follows:

(Equation 8) $$I=\frac{n}{W}*\frac{\sum_{i}\sum_{j}w_{ij}\left(x_{i}-\bar{x}\right)\left(x_{j}-\bar{x}\right)}{\sum_{i}{\left(x_{i}-\bar{x}\right)}^{2}}$$

In this formula, *n* represents the total number of pixels, *x*_*i*_ denotes the LST value of the i-th pixel, $\bar{x}$ is the average LST value of the region, *w*_*ij*_ is the spatial weight between adjacent pixels *i* and j, and $W=\sum_{i,j}w_{ij}$ is the normalization coefficient.

Additionally, to capture the specific distribution of hotspot regions, the Getis-Ord Gi∗ hotspot analysis method is introduced to perform a local clustering significance test on the pixels. Based on their Z-values, high-temperature hotspots (Z > 1.96, *p* < 0.05) and cold spots are identified, providing a basis for hotspot boundary identification.

#### Modeling thermal environmental drivers and XGBoost-SHAP model

To quantify the key factors influencing LST changes and reveal their mechanisms, an XGBoost regression model was used to model various natural, social, and land factors.^96^^,^^97^ The prediction target form of XGBoost is as follows:

(Equation 9) $$\hat{y}=\sum_{k=1}^{k}f_{k}\left(x\right),f_{k}\in F$$

where *x*=(*x*_1_,*x*_2_, …,*x*_*n*_) are the input features, including normalized NDVI, NDBI, albedo, nighttime light index, population density, building coverage, altitude, and other variables. represents the *k-th* regression tree. *F* denotes the set of tree models. In this study, the XGBoost models were fitted separately for daytime and nighttime summer LST in 2020 at the pixel level across the BTHUA. Therefore, the modeling scope is cross-sectional and spatial, aiming to explain the spatial heterogeneity of LST in a representative year rather than the interannual evolution of LST from 2000 to 2024. Accordingly, the driver analysis in this section is not intended to provide a temporally matched explanation for the full 2000–2024 heatwave evolution, but rather to identify the dominant spatial associations underlying daytime and nighttime thermal patterns in a representative summer background year. The model was tuned using cross-validation and then interpreted using SHAP values.

To enhance the model’s interpretability, the SHapley Additive exPlanations (SHAP) value method was further applied to calculate the marginal contribution of each feature to the model output, with the mathematical expression given by^98^:

(Equation 10) $$\phi_{i}\left(f,x\right)=\sum_{S\subseteq F\setminus \left\{i\right\}}\frac{|S|!\left(|F|-|S|-1\right)!}{|F|!}\left[f_{S\cup \left\{i\right\}}\left(x_{S\cup \left\{i\right\}}\right)-f_{s}\left(x_{s}\right)\right]$$

In the formula, ϕ_*i*_ is the SHAP value of feature *i*, representing its contribution to the model prediction of LST. *S* is the feature subset. By ranking SHAP values and examining their distributions, we identify which variables contribute more strongly to the model output and how their associations with predicted LST vary across value ranges. These results improve model interpretability, but they should be understood as model-based associations rather than direct evidence of causal mechanisms.

### Quantification and statistical analysis

All quantitative analyses were performed at the pixel level using valid observations after quality control. Annual summer LST values were calculated as pixel-level seasonal means from MODIS observations during June-August, and annual spatial summaries were then derived from all valid pixels within the BTHUA. Heatwave events were identified using the annual summer 90th-percentile LST threshold and a minimum duration of three consecutive observations. Heatwave frequency, average duration, intensity, and severity were calculated as event count, mean event length, mean exceedance above the threshold, and cumulative exceedance above the threshold, respectively.^93^^,^^94^^,^^99^ Temporal trends in annual LST and Moran’s I were assessed using two-sided Mann–Kendall trend tests, and trend magnitudes were estimated using Theil–Sen slopes. Spatial autocorrelation significance was evaluated using Moran’s I permutation tests, and local hotspots/coldspots were identified using Getis-Ord Gi∗ statistics with Z > 1.96 and *p* < 0.05 as the criterion for statistical significance.^98^^,^^100^^,^^101^^,^^102^^,^^103^ For boxplots, center lines indicate medians, boxes indicate interquartile ranges, whiskers extend to 1.5 times the interquartile range, and points indicate outliers. Unless otherwise specified, the figures do not present mean ± SEM error bars; instead, distributional summaries are defined in each relevant figure legend. Statistical annotations in figures are defined as follows: ns, *p* ≥ 0.05; ∗, *p* < 0.05; ∗∗, *p* < 0.01; ∗∗∗, *p* < 0.001.
